# A novel approach to systematically collect critical chloride contents in concrete in an open access data base

**DOI:** 10.1016/j.dib.2019.104675

**Published:** 2019-10-23

**Authors:** C. Boschmann Käthler, U.M. Angst, A.M. Aguilar, B. Elsener

**Affiliations:** a ETH Zurich, Institute for Building Materials (IfB), CH-8093, Zurich, Switzerland; b University of Cagliari, Department of Chemical and Geological Science, I-09100, Monserrato, CA, Italy

**Keywords:** Carbon steel, Concrete, Steel reinforced concrete, Critical chloride content, Pitting corrosion

## Abstract

A living data collection providing critical chloride contents for steel corrosion in concrete (C_crit_) is presented. The C_crit_ values were measured on samples taken from engineering structures. This approach allows to overcome the well-known limitations of testing laboratory samples. The data are available in a public data repository. Currently, 46 C_crit_ are reported (measured on four different structures). The database will continuously be updated. The database includes information about the structure, material properties, and local condition of the steel-concrete interface. For possible applications of this database and discussion refer to the related research article in Corrosion Science.

**Table:** Specifications Table

Subject area	*Civil engineering and materials science*
More specific subject area	*Chloride-induced steel reinforcement corrosion in concrete*
Type of data	*Table and figure*
How data was acquired	*Concrete cores were drilled from structures and tested with a defined protocol in the laboratory* [1].
Data format	*The data of the tests are C*_*crit*_*as well as supplemental information (material properties, local conditions, etc.). Raw and analyzed*
Experimental factors	*Material properties (such as w/b-ratio, cement type, steel microstructure …), exposure conditions, type of structure and structural element*
Experimental features	*Exposure of reinforced concrete samples from engineering structures to chloride solution until corrosion initiates. Measuring the chloride content at reinforcement level. Characterizing concrete and reinforcing steel by various methods including microscopic techniques.*
Data source location	*Reinforced concrete engineering structures from various geographic locations, primarily in Switzerland but also other countries*
Data accessibility	*The data is available in a public repository: A data collection of critical chloride contents for steel corrosion in concrete* [2]*, URL:*https://doi.org/10.3929/ethz-b-000282371
Related research article	*C. Boschmann Käthler, U.M. Angst, A.M. Aguilar, B. Elsener “A systematic data collection on chloride-induced steel corrosion in concrete reveals the mechanism of corrosion initiation and improves service life modelling”, Cor Sci 157, 2019,*https://doi.org/10.1016/j.corsci.2019.06.008 [3]

**Table:** 

**Value of the Data** • This data is important for understanding and predicting corrosion of steel in concrete. The reported data was obtained with a single, well-defined method and contains all relevant parameters on corrosion susceptibility. • Civil engineers can use the data as input parameter for service life design. • The data can be used by corrosion scientists to enhance the fundamental understanding of corrosion initiation. • Using specimens from engineering structures ensures realistic conditions at the steel-concrete interface. While this is crucial, it cannot be achieved in experiments with specimens produced in the laboratory.

## 1 Data

### 1.1 Online repository and data structure

The data is available online in the public repository [2].

At present, this file contains C_crit_ and supplemental information from in total 46 tested samples (taken from 4 reinforced concrete structures). This file will be continuously updated in order to make new data accessible to the community. The file in the repository indicates the date of publication.

The database contains the information summarized in Table 1. This will be described in more detail in section 2. Abbreviations used in the database are explained in Table 2.

**Table 1:** Summary of information reported in the data file. For further explanation, see Chapter 2.2 to 2.5

Reported information		Remarks
Sample identification number		Each tested sample has an identification number to permit unambiguous assignments (Fig. 1 and Chapter 2.2)
Engineering structure information	Year of construction	based on documents about structure
	Location	Geographic location of the structure
	Meters above sea level (m a.s.l.)	according to geographic location
	Type of structure	e.g. tunnel, bridge, etc.
	Element	The type of structural member from which the samples were taken (e.g. tunnel wall, tunnel ceiling, abutment wall, bridge deck/slab, etc.) (compare Fig. 1)
	Exposure class	According to EN Standard EN 206-1
	Area of sampling (m^2^)	Size of area (in m^2^ of concrete surface), within which drilling cores were taken. This area applies to each sample series within an element (compare Fig. 1)
Concrete information	Cover depth (mm)	Cover depth measured on the drilling cores immediately after sampling from the structure
	Cement type	based on available documentation or characterization of obtained samples
	w/b-ratio	based on available documentation or characterization of obtained samples
	aggregates	based on available documentation or characterization of obtained samples
	Compressive strength (MPa)	Compressive strength of concrete according to documentation of structure or measured on additional samples
	Concrete resistivity (Ωm)	In wet conditions, measured during the corrosion test in the laboratory
	Non-carbonated cover depth (mm)	Distance from the carbonation front (phenolphthalein spray test) to the steel, measured on the drilling cores after splitting the sample
	pH of pore solution at level of reinforcement	Measured on the drilling cores after splitting the sample as described in [4, 5]
Steel information	Steel bar diameter (mm)	measured on the sample
	Orientation of reinforcement in the structure	Horizontal (H) or vertical (V), see Figure 2
	Type of steel	Qualitative information about steel, such as chemical composition, and rib geometry
	Microstructure near surface	in the zone close to the surface
	Steel potential information (mV vs. Ag/AgCl_sat_)	Measured in the laboratory at the beginning of chloride exposure testing, before corrosion initiation, and at the end of the corrosion test
Critical chloride content (% by mass of concrete and of binder)		Acid-soluble chloride content at level of reinforcement
Steel-concrete interface (SCI)	Visually observable characteristics at the initiation spot on the concrete	Irregularities such as pores, cracks, tie wires, etc. at the SCI
	Visually observable characteristics elsewhere on the concrete	Irregularities such as pores, cracks, tie wires, etc. at the SCI, but at locations where no corrosion initiated
	Visually observable characteristics at the initiation spot on the steel	This includes mainly if the initiation spot is on a rib, adjacent to a rib, or between the ribs.
	Location of corrosion initiation with respect to the rebar orientation in the structure	See Fig. 2
Laboratory		Name of the laboratory that conducted the test (chloride exposure of drilling cores in the lab and analysis)

**Table 2:** Abbreviations used in the data collection.

Abbreviation	Meaning
Orientation of reinforcement in the structure	
V	vertical
H	horizontal
Characteristics on the concrete interface	
N	nothing
A	air void (<1 mm)
CA	coarse air void (>1 mm)
A or CA	initiation spot at A or CA
A-E or CA-E	initiation spot at the edge of A or CA
D	deposit
CE	crack from steel to exposure side
CR	cracks at SCI at ribs
W	tie wire
Characteristics at the steel interface	
R	rib
aR	adjacent to rib
bR	between ribs
Orientation of corrosion initiation with respect to structure	
S	side
U	upper side
L	lower side
Others	
NR	not reported
PC	Portland cement
SCI	steel-concrete interface

## 2 Experimental design, materials and methods

### 2.1 Overview

Concrete cores (diameter = 150 mm, height: at least 50 mm behind the cover depth) were drilled from reinforced concrete structures. Each core contained a centrally located reinforcing steel bar, which, at the time of sampling, was not yet corroding (tested on-site with half-cell potential mapping). The samples were transferred to the laboratory and subjected to a preparation protocol, which included establishing electrical connections to the rebar for measuring purposes as well as coating lateral faces of the concrete sample to ensure 1-dimensional chloride ingress during the laboratory test and to avoid undesired rebar end effects (corrosion testing artefacts). The corrosion state was monitored by means of electrochemical measurements. Upon corrosion initiation, the chloride content in the concrete at level of reinforcement was measured to determine the C_crit_ (acid-soluble total chloride content measured with potentiometric titration according to Swiss Standard [6] or an equivalent international standard). Additionally, the sample was split and investigated for a number of material properties described in more detail below (Section 2.4, 2.5, 2.7). The complete experimental protocol, including all steps from taking the samples on the structure to testing them in the laboratory, is described in Ref. [1]. In addition, data from laboratory specimens that were prepared with the same experimental protocol as the samples from structures will also be reported.

We consider it a particular advantage that all the C_crit_ values reported here were determined with one single method. This permits to narrow the influence of the test method itself, which, due to the many different approaches used in the literature, is one of the main factors complicating the interpretation and comparability of literature results [7].

### 2.2 Sample labeling and numbering

**Fig. 1:**
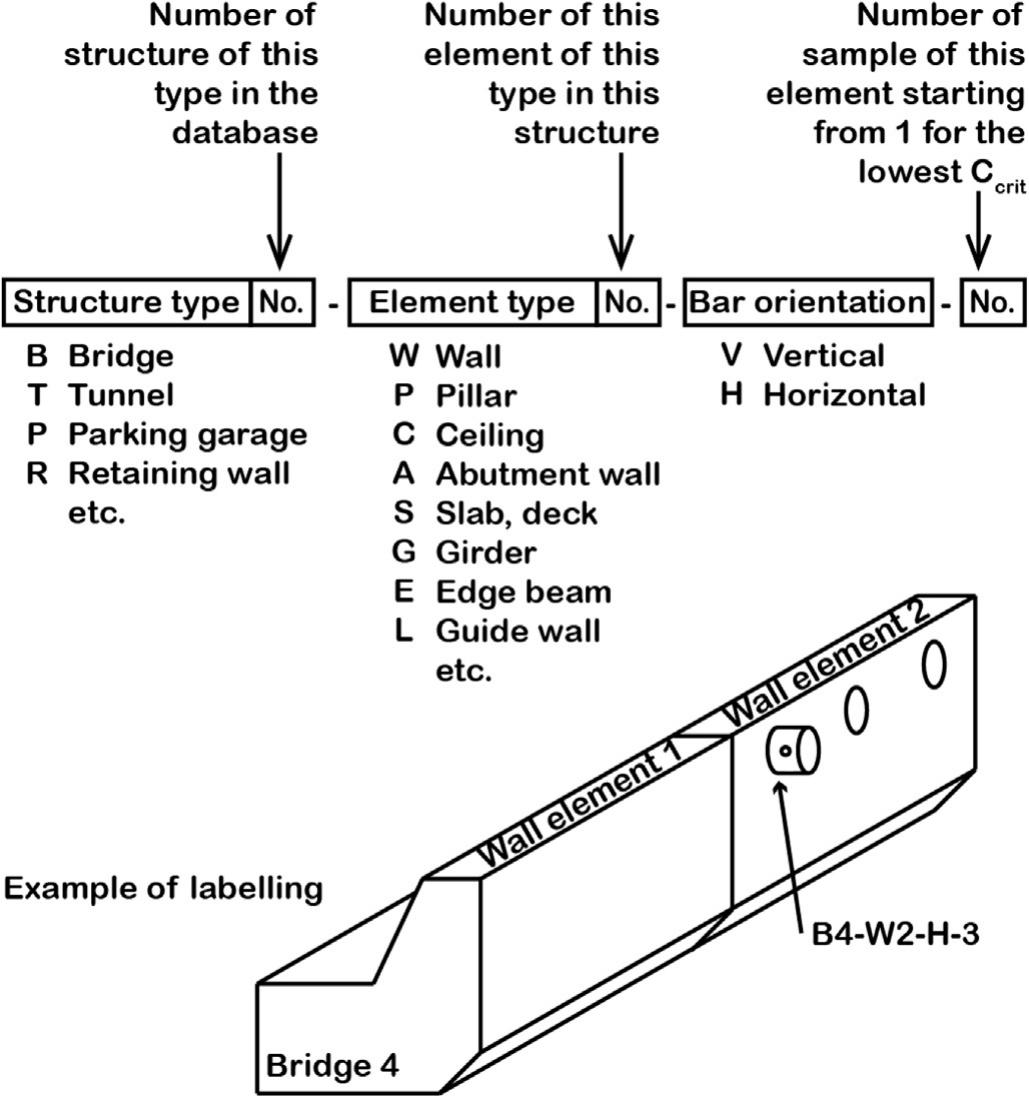
Structure of labelling of each specific sample.

Each tested sample received an identification number to permit unambiguous assignments. The samples were labeled and numbered as schematically described in Fig. 1. The labels indicate the type of the engineering structure, the type of element within the structure, and the steel bar orientation within the structure (namely H and V). Numbers indicate the number of the structure in the database, the number of an element of a given type within the structure (sometimes, samples were taken from different elements within one structure), and finally, the number of the sample within an element. These last digits in the labeling string (Fig. 1) indicate the sample number, sorted according to the final result of C_crit_ (from lowest to highest). The highest sample number within an element is equal to the number of samples tested within this element.

Fig. 1 shows an example (B4–W2–H-3). Here, B4 means that this is the forth bridge in the data collection; within this bridge, samples were taken from different wall elements, and B4–W2–H-3 is the third sample taken from the second tested wall element in the bridge.

### 2.3 Engineering structure information

As shown in Table 1, the data collection contains information about the engineering structure. Generally, structures older than 20 years and in chloride-exposure environments were selected. Drilling cores were taken from non-corroding, non-carbonated (at level of reinforcement) areas which preferably were easily accessible.

The geographic location of the structure, type of structure, as well as the specific element of the structure are documented. Information related to the structure (year of construction, meters above sea level, exposure class acc. to EN standard 206-1) as well as information related to the sampling area (size and height above ground) are also given in the data collection.

### 2.4 Concrete properties

The cover depth of the reinforcement was measured directly after drilling the sample from the structure.

Information about cement type and mix proportions were obtained either through microscopy analysis or according to documentation on the structure, in case the latter was available. This includes w/b-ratio, cement type, and aggregate details (type and size).

The electrical concrete resistivity was quantified after splitting the samples, that is, at the end of the laboratory test when the concrete was in wet conditions (but below saturation). Concrete cubes with an approx. dimension of 50 mm × 50 mm x 50 mm were used for resistivity measurements. Resistivity was measured with two steel plate electrodes according to the recommendation from RILEM TC-154 [8].

After splitting the sample, the carbonation depth was evaluated with help of the phenolphthalein spray test, namely by spraying on the freshly broken concrete surface in the cover depth [1]. The indicator changes its color above pH 8.5 to pink (below this pH it is colorless). The distance from the carbonation front to the exposed side of the reinforcing steel is given in the database (non-carbonated cover depth). The pH of the pore solution at level of reinforcement is measured by a method described in [4,5]. It consists basically in drilling a small cavity in the concrete surface, filling it with distilled water, covering it to avoid carbonation of the solution, and measuring the pH in the solution in the cavity once per day over a week (until a stable value is achieved).

### 2.5 Steel properties

**Fig. 2:**
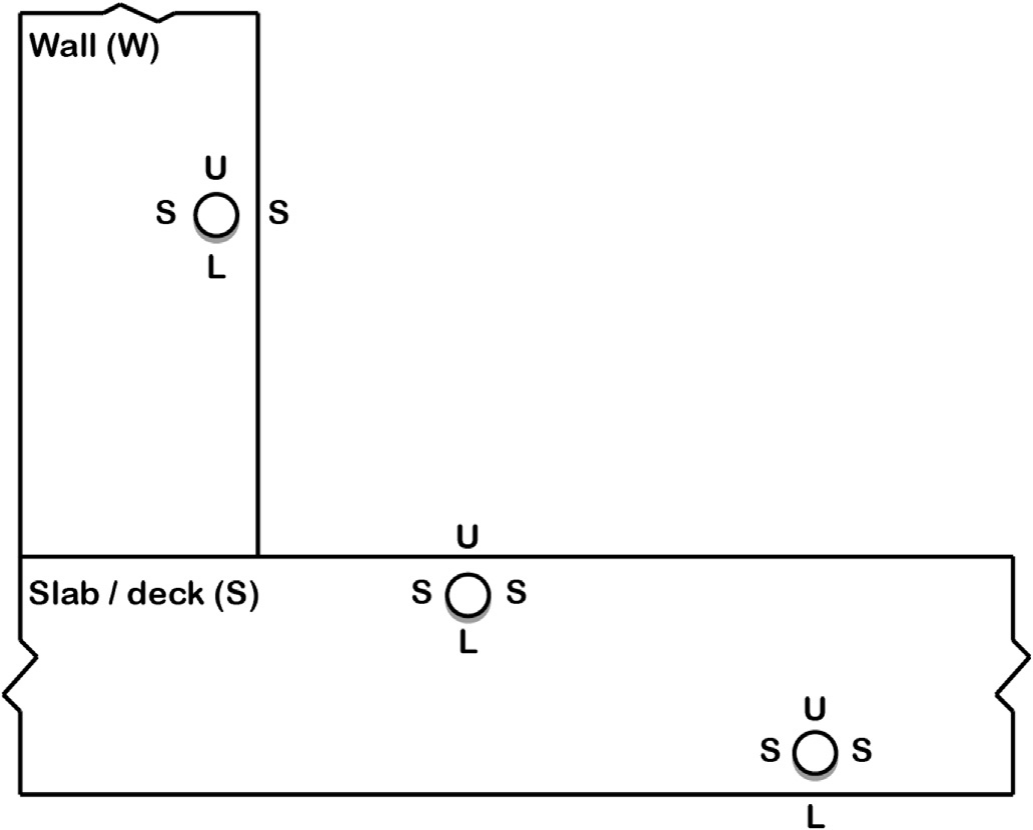
Labelling of orientation of exposed side of horizontal reinforcement. The grey areas below the rebar represent the region of plastic settlement, where special conditions at the SCI might occur [10].

The diameter of the reinforcement in the drilling cores was measured. Moreover, the orientation of the reinforcement in the structure and with respect to the chloride exposure side is documented (Fig. 2). Reinforcement is either vertically (V) or horizontally (H) oriented. For horizontal bars, it is well-known that the concrete microstructure of the steel-concrete interface differs between upper and lower sides as well as the vertical parts of the cross-sectional circumference [9,10]. Thus, the data collection contains this information, with help of the labelling terminology illustrated in Fig. 2.

Information about the type of steel is given according to structure documentation. This includes the chemical composition of the steel (carbon steel or alloyed steel), the existence of ribs (smooth or ribbed surface), and its microstructure. The steel microstructure of the used reinforcement is analyzed with polished and etched (3% Nital for 3–5 s) sections under an optical microscope [11].

The steel potentials were monitored with an automated data-logger and a Ag/AgCl_sat_-reference electrode [1]. In the data collection, the potentials (vs. Ag/AgCl_sat_) are documented at the beginning of exposure, immediately before corrosion initiation and at the end of the corrosion test.

### 2.6 Critical chloride content

The acid-soluble chloride content corresponding to corrosion initiation (C_crit_) was measured at level of the reinforcement, referred to mass of concrete. To convert it to mass of cement, we used an image analysis method [12] to determine the amount of cement in the actual concrete sample for the chloride test, because this value can differ significantly from a bulk cement content and from the assumption of a constant cement content (*e.g.* according to Swiss Standards [6]: 300 kg of cement per m^3^ concrete). Both values (viz. the C_crit_ with an assumed cement content of 300 kg of cement per m^3^ concrete and the C_crit_ with the measured cement content in each specific sample) are reported in the data collection.

### 2.7 Steel-concrete interface

**Fig. 3:**
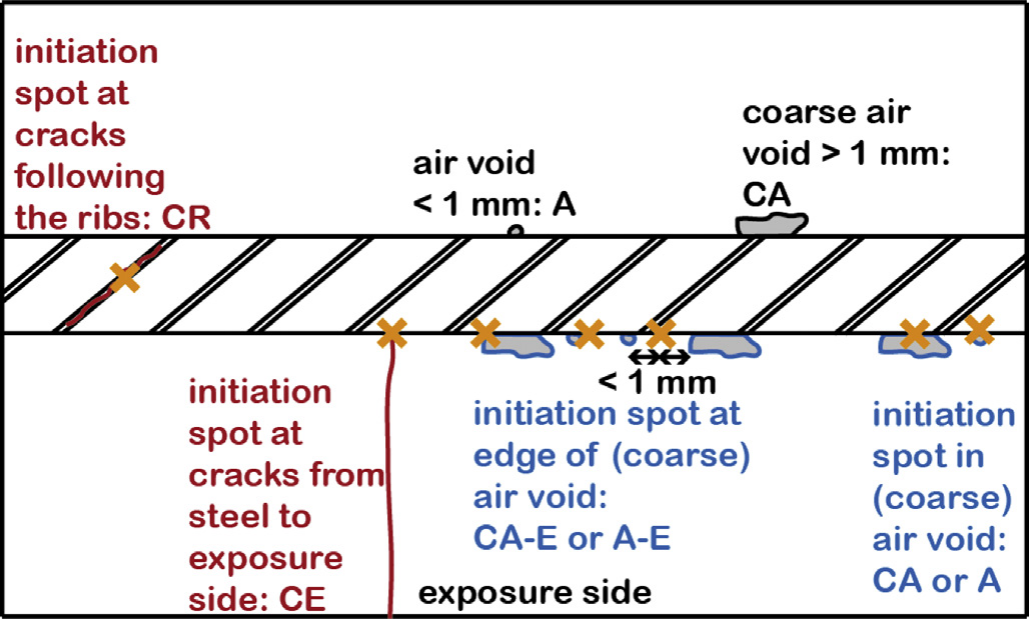
Illustration of different kinds of air voids and cracks.

The local conditions [9] at the corrosion spot and elsewhere at the steel-concrete interface were visually inspected over the whole steel-concrete interface (with the naked eye and with an optical microscope (max. magnification 4×)). The data collection reports the presence of any visually detectable irregularities in the concrete at the very site of corrosion initiation. Frequent examples are air voids (A), cracks (C), deposits (D), and tie wires (W) at the concrete side of the interface. The feature air void (A) and cracks (C) are distinguished in Fig. 3: (A) is divided in coarse air voids (>1 mm, CA) and air voids (<1 mm, A). It is documented, whether the corrosion spot is located within the air void (CA or A, respectively), or at the edge of an air void (CA-E or A-E, respectively). Here, “at the edge” means that the initiation spot is not where the air void touches the rebar, but in the cement paste in a region within max. 1 mm distance from the air void. The cracks are divided in cracks which reach from the steel to the exposure side in the lab (CE), or small cracks at the steel-concrete interface (CR), mostly located at the ribs. Note that cracks reaching the exposure side strongly affect the transport of chlorides through the cover and the related C_crit_ values cannot be directly compared to C_crit_ measured in samples free from cracks through the cover. Thus, data from CE-samples are in the data collection reported in parentheses.

Moreover, the data collection reports the presence of irregularities at locations that do not correspond to the corrosion initiation site. For instance, in virtually all specimens there are air voids present at the SCI, but only in some cases do these correspond to the location of corrosion initiation.

The data collection contains information regarding the location of corrosion initiation with respect to the rebar topography, namely if the corrosion spot was located on the steel surface on a rib (R), adjacent to a rib (aR) and between two ribs (bR). Here, aR means that the corroding spot was right at the very edge of the rib, whereas bR means that the corroding spot was further away from this edge, located somewhere on the “plain” surface of the rebar.

For horizontally oriented reinforcement, the location of the corrosion spot is documented in relation to the structure. Fig. 2 depicts the orientation labelling of an initiation spot.
